# Clear Aligner Therapy in Adults With Stage II-IV Periodontitis: A Narrative Review

**DOI:** 10.7759/cureus.103095

**Published:** 2026-02-06

**Authors:** Sunil Kumari, Greeshma Gothankar, Nithin KV, Sangeeta Rai, Shailendra Singh Rana, Ashutosh Dixit

**Affiliations:** 1 Orthodontics and Dentofacial Orthopedics, All India Institute of Medical Sciences, Rishikesh, Rishikesh, IND; 2 Orthodontics and Dentofacial Orthopedics, All India Institute of Medical Sciences, New Delhi, New Delhi, IND; 3 Orthodontics, Mahatma Gandhi Mission Dental College and Hospital, Mumbai, IND; 4 Prosthodontics, All India Institute of Medical Sciences, Rishikesh, Rishikesh, IND; 5 Periodontics, All India Institute of Medical Sciences, Rishikesh, Rishikesh, IND; 6 Dentistry, All India Institute of Medical Sciences, Bathinda, Bathinda, IND; 7 Dentistry, All India Institute of Medical Sciences, Rishikesh, Rishikesh, IND

**Keywords:** clear aligner therapy, interdisciplinary treatment, orthodontic treatment, pathologic tooth migration, periodontitis

## Abstract

Stage II-IV periodontitis is frequently associated with pathologic tooth migration (PTM), occlusal instability, and complex restorative demands requiring coordinated interdisciplinary care. Adult patients with periodontitis increasingly request clear aligner therapy (CAT); however, its role within structured periodontal-orthodontic treatment pathways remains incompletely defined. This narrative review synthesized available clinical evidence on the use of clear aligner therapy in adults with stage II-IV periodontitis and outlined practical principles for interdisciplinary management. A literature search was conducted using PubMed, Scopus, Embase, and Google Scholar. Eligible clinical reports included adults diagnosed with periodontitis or a reduced but stable periodontium who were treated with clear aligner therapy as part of an interdisciplinary protocol and reported periodontal and/or occlusal outcomes. Nine clinical studies met the eligibility criteria, including case reports, small case series, and one prospective pilot study involving stage IV periodontitis. Across all reports, clear aligner therapy was initiated only after completion of comprehensive periodontal infection control and enrollment in regular supportive periodontal care (SPC). In stable stage II-IV patients, clear aligner therapy improved occlusal relationships and pathologic tooth migration while maintaining periodontal parameters. No study demonstrated periodontal superiority over fixed appliances. Clear aligner therapy appears to be a viable orthodontic option when integrated into a structured interdisciplinary protocol, although higher-quality prospective studies are required.

## Introduction and background

Periodontitis is a prevalent chronic inflammatory disease and a leading cause of tooth loss. Stage III and IV periodontitis often lead to secondary occlusal trauma, pathologic tooth migration (PTM), bite collapse, and esthetic issues [1-4]. Adult patients increasingly seek orthodontic correction for these sequelae. A structured approach to managing stage II-IV periodontitis involves risk factor control, infection control, corrective periodontal therapy, and supportive periodontal care (SPC). Orthodontic treatment is no longer contraindicated in periodontitis patients, provided inflammation is controlled and patients are on long-term maintenance [1,5,6]. Orthodontic tooth movement can redistribute occlusal forces, resolve traumatic occlusion, improve plaque control access, and facilitate prosthetic rehabilitation [4,6]. This collaborative approach is termed "ortho-perio" care. Historically, most evidence on orthodontics in periodontitis involved fixed appliances. Studies suggest that in well-maintained periodontitis patients, fixed appliance orthodontics can maintain or improve periodontal conditions, especially with regenerative techniques and careful biomechanics [6]. However, fixed appliances can increase plaque retention and transiently worsen gingival indices, which is a concern for patients with prior periodontal breakdown [1,7,8]. Clear aligner therapy (CAT) offers an esthetic, removable alternative for adult orthodontics [5]. For periodontally compromised adults, CAT may offer advantages such as easier oral hygiene, reduced soft tissue irritation, and digital planning for movements within a reduced alveolar bone [1,5,9-11]. Conversely, aligners and attachments can create plaque-retentive areas, and repeated insertion/removal may transmit unfavorable forces to mobile teeth. Thus, CAT's net periodontal effect in stage II-IV periodontitis needs specific investigation [12].

A prior review by dos Santos et al. [13] and Rossini et al. [14] discussed CAT's impact on periodontal health, focusing on plaque and gingival indices, microbiological shifts, and mucogingival conditions in general orthodontic samples, briefly touching on advanced periodontitis and PTM. Since then, new clinical reports described CAT in stage II-IV periodontitis, often post-regenerative surgery and within interdisciplinary plans. Given the rising demand for esthetic treatment in periodontitis patients, a focused synthesis on integrating clear aligners safely into stage II-IV ortho-perio care is needed. This review summarizes clinical reports of CAT in adults with stage II-IV periodontitis, emphasizing on PTM and post-regenerative cases. It highlights common principles of timing, biomechanics, and SPC, and compares findings with broader literature to offer practical interdisciplinary management recommendations [2,3,15,16].

## Review

Methodology

This narrative review aimed to synthesize clinical evidence on CAT in adults with stage II-IV periodontitis within an interdisciplinary framework, deriving practical management principles. Due to the largely case-based and pilot nature of existing literature, a qualitative synthesis was deemed most appropriate.

Information Sources and Search Strategy

**Figure 1:**
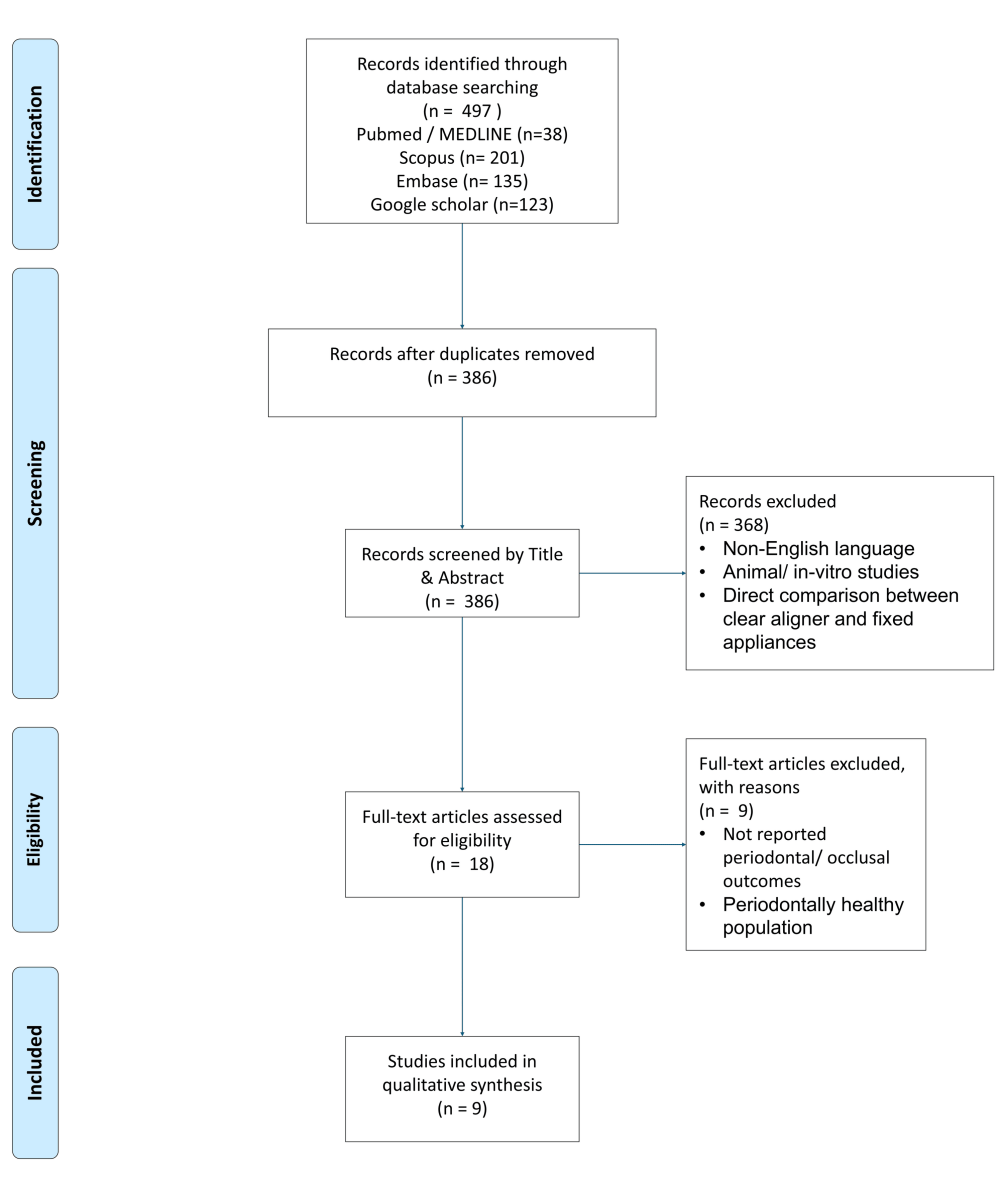
Study selection process

Electronic literature searches were performed in PubMed/MEDLINE, Scopus, Embase, and Google Scholar up to December 2025 (Figure 1). The search strategy combined terms for clear aligners, periodontal disease, and orthodontic treatment. The complete search strategies are detailed in Appendix 1.

An initial search yielded 497 records across PubMed (38), Scopus (201), Embase (135), and Google Scholar (123).To ensure comprehensive coverage, a manual hand search was performed to identify additional relevant studies not captured in the electronic search. All results were imported into Mendeley reference management software for organization and deduplication. This process left 386 unique records for screening.

Eligibility Criteria

The review included English-language, peer-reviewed studies involving adult patients with stage II-IV periodontitis or a stable reduced periodontium treated with clear aligner therapy. Studies were required to report periodontal and/or occlusal outcomes and to describe treatment delivered within an interdisciplinary periodontal-orthodontic care framework. Eligible study designs included systematic reviews, clinical studies, case series, and case reports.

Exclusion criteria included the use of clear aligners solely for retention purposes, enrollment of participants without periodontal disease, animal or in vitro study designs, and studies in which the primary objective was a comparative evaluation of clear aligners versus fixed orthodontic appliances.

Study Selection and Data Extraction

Titles and abstracts were screened for relevance. Potentially eligible full texts were assessed against the inclusion criteria. Nine clinical articles meeting all criteria form the primary evidence base. Key data extracted for each report included patient demographics, periodontal diagnosis/stage, presence of pathologic tooth migration, periodontal phase details, timing of CAT initiation, aligner system/biomechanics, SPC protocol, treatment duration, and reported outcomes. Follow-up data were recorded when available.

Synthesis Approach

Due to high variability in study design, sample size, and outcomes, a qualitative, descriptive synthesis was performed instead of quantitative pooling or formal risk-of-bias assessment. Findings were qualitatively grouped as follows: CAT after periodontal stabilization in stage II-III/chronic periodontitis, CAT after regenerative surgery in stage IV periodontitis with pathologic tooth migration, and common interdisciplinary principles and SPC frameworks. Themes in timing, case selection, biomechanics, and maintenance were identified to propose a practical stepwise protocol.

Overall picture of the available clinical evidence

The nine clinical reports reviewed demonstrate that CAT can be integrated into managing adults with stage II-IV periodontitis, provided strict periodontal preconditions are met and treatment follows a structured ortho-perio pathway. Patients were typically middle-aged adults with a generalized periodontitis history, a stable reduced periodontium at referral, and chief complaints including pathologic tooth migration, anterior spacing/flaring, deep overbite, and occlusal instability. CAT was always part of an interdisciplinary approach, initiated only after infection control, corrective periodontal therapy, and enrollment in SPC. A consistent pattern emerged: when CAT started in a stable reduced periodontium with low plaque/bleeding scores, periodontal parameters remained stable or modestly improved, while significant functional and esthetic orthodontic changes were achieved. Probing pocket depths (PPD) and radiographic bone levels showed favorable trajectories, similar to fixed-appliance treatment in periodontitis patients.

Stage II-III or Chronic Periodontitis: Periodontal Safety and Occlusal Benefits of CAT

In patients with stage II-III or chronic periodontitis post-non-surgical therapy, CAT primarily corrected pathologic tooth migration, deep overbite, extrusion, and crowding in a reduced periodontium [17,18]. CAT began only when full-mouth plaque/bleeding scores were below 15%-20%, no tooth was to be moved, had residual probing depths >4 mm, and mobility was reduced to grade I or less [1,13]. Under these conditions, aligners provided meaningful orthodontic benefits without further breakdown [10,17,19,20]. Functionally, CAT intruded extruded incisors, retracted flared anterior teeth, closed diastemata, and resolved crowding. One report noted an 83% reduction in Peer Assessment Rating score, indicating substantial occlusal and alignment improvement while maintaining periodontal support [10]. Others showed traumatic occlusion elimination and improved overjet/overbite in patients with periodontitis-induced migration/spacing. Periodontally, these stage II-III/chronic cases showed stability. Shallow, non-bleeding sites remained stable over 9-18 months of treatment. Detailed measurements sometimes showed small gains in clinical attachment loss (CAL) and reductions in infrabony defect dimensions. Importantly, no reports indicated new deep pockets or sudden attachment loss attributable to CAT once inflammation was controlled. These studies suggest that in stage II-III or chronic periodontitis, CAT's main value is functional and esthetic, restoring axial inclinations, incisal relationships, and crowding to improve self-care access and simplify restorative planning, while periodontal health remains stable with appropriate SPC. It complements, rather than replaces, high-quality periodontal therapy [5,10,12,13,17]. Quantitative data from two severe periodontitis cases with 12-month follow-up showed mean full-mouth probing depths in the low 2-3 mm range, and mean CAL varying by less than 0.3 mm, confirming periodontal stability under three-monthly SPC while occlusal and esthetic goals were met [4].

Table 1 presents the characteristics and periodontal-orthodontic outcomes of clear aligner therapy in patients with periodontitis, and Table 2 presents the changes in clinical and radiographic periodontal values reported in the included studies.

**Table 1:** Characteristics and periodontal-orthodontic outcomes of clear aligner therapy in patients with periodontitis CAT: clear aligner therapy, SPC: supportive periodontal care, SRP: scaling and root planning, BOP/BoP: bleeding on probing, PPD: probing pocket depth, CAL: clinical attachment level/loss, PI: plaque index, FMPS: full-mouth plaque score, FMBS: full-mouth bleeding score, PTM: pathologic tooth migration

Author	Study design	Patient demographics	Periodontitis stage	Timing of CAT initiation	Aligner system/biomechanics	SPC protocol	Treatment duration	Reported outcomes
Raphaelli Nahás et al. [4]	Case report	Two male participants (43-year-old and 62-year-old)	Stage III, grade B periodontitis	After 12 months of periodontal maintenance post-SRP	Clear aligner system; 23 upper, 35 lower aligners for first phase; light-curing resin attachments; worn 22 hours/day; changed every 14 days	Periodontal maintenance and oral hygiene instruction every three months post-treatment, continued during orthodontic treatment	12 months for the first phase	Stable periodontal parameters and successful orthodontic tooth movement, achieving significant occlusal improvement in adult patients with reduced periodontium
Ronsivalle et al. [5]	Case report	50-year-old female patient	Stage III, grade B	After periodontal stabilization, when BOP < 10% and PI < 20% achieved (6 months after the start of periodontal treatment)	Invisalign® system with ClinCheck® software for digital planning; biomechanics defined "a priori" for controlled, light forces; emphasizes digital orthodontic setup for strategic planning and predictability; programmed movement per aligner; 0.15 mm, to avoid less stress on dentition and alveolar ridge crest	The goal was to achieve BOP < 10% and PI < 20% before starting CAT; periodontal treatment involved full-mouth SRP in two sessions	Less than one year	Successful achievement of functional, periodontal, and aesthetic orthodontic goals using clear aligners with effective force control after periodontal stabilization
Zhang et al. [10]	Case report	24-year-old female patient	Severe chronic periodontitis (inactive)	After six months of systemic periodontal treatment	Angel Aligner system; patient wore each aligner ~22 hours/day, changed every 14 days; treatment in initial and refinement stages; designed for 1.9-2.4 mm anterior incisor intrusion, 14-15 degrees labial crown movement of lingually inclined incisors; optimized and conventional attachments, bite ramps used; gentle instantaneous force applied; movement: 0.1 mm at each step	Periodontal maintenance arranged every three months during orthodontic treatment	22 months of active treatment	Successful orthodontic treatment maintaining good periodontal health and inactive periodontitis status; clear aligners offer advantages for patients with periodontal disease
Turatti et al. [17]	Case report	43-year-old female patient	Adult periodontitis, stage not reported	After scaling and root planning	Invisalign® system; 15 upper, 10 lower aligners; aligners changed every 15 days; light, continuous force; attachments used for sectional intrusion; movement rate: 0.025 mm aligner	Scaling repeated every eight weeks during orthodontic treatment	11 months	Effective orthodontic intrusion reduced infrabony defects and improved periodontal health, achieving successful alignment of extruded incisors using Invisalign with light, continuous forces
Lee et al. [18]	Case report	One male patient (50 years old) detailed; three patients total sampled	Chronic periodontitis with pathologic tooth migration	After periodontal treatment	Thermoplastic clear hard type polymer; 0.5 mm then 0.75 mm thickness; teeth moved not more than 1 mm per appliance; new appliances every three weeks; worn all day except meal times	After a series of case-related periodontal therapy; no specific ongoing SPC protocol mentioned during CAT	Not specified	Effective for aesthetic restoration and stabilization of periodontally compromised teeth, with clinical and radiographic reductions in periodontal parameters
Stańdo-Retecka et al. [19]	Case report	46-year-old female patient	Advanced periodontitis, stage IV, grade C	One year after the beginning of periodontal treatment	Clear Aligner; three sets of six adjusted thermoplastic aligners; first set changed after six weeks, subsequent aligners replaced every eight weeks	Periodontal maintenance visits scheduled every two months, including examination and professional plaque removal.	Five months	Complete resolution of periodontal inflammation, improved aesthetics, and stable occlusion achieved at three-year follow-up through a multidisciplinary approach
Tietmann et al. [21]	Retrospective analysis	10 patients; mean age: 51.5 years; six female and four male patients	Stage IV (type 2) periodontitis with intrabony defects requiring regenerative surgery	Mean of 4.5 months after periodontal regenerative surgery, depending on tooth mobility	Clear aligners used in all patients; low forces and moments for tooth movements; attachments bonded as needed; aligners worn full-time except meals/oral hygiene; changed every two weeks	SPC at an interval of four weeks up to three months during the whole duration of orthodontic treatment	8-12 months (mean: 12.6 months)	Successful orthodontic alignment and stable periodontal conditions following regenerative periodontal surgery, with significant improvements in intrabony defects and PPD reduction
Ravera et al. [22]	Prospective pilot study	21 patients enrolled, 11 underwent treatment; 4 male and seven female patients (mean age: 50.7 ± 12.4 years)	Stage IV, grade B periodontitis with PTM	9-12 weeks after completion of periodontal non-surgical and surgical treatment	Invisalign® system; light force application; focus on reducing posterior occlusal contacts	Not reported explicitly for ongoing SPC protocol during CAT, but states "proper oral hygiene is maintained" and "plaque-induced inflammation be completely suppressed before the application of orthodontic forces"	Approximately 19 months	Overall stability of periodontal indices and successful orthodontic treatment outcomes, with statistically significant reductions in PPD and CAL
Almagrami et al. [23]	Case report	53-year-old female	Localized stage III, grade B	After initial periodontal therapy and re-evaluation (approximately one month)	Invisalign®; 56 maxillary and 88 mandibular aligners; seven-day change interval; ≥22 hour/day wear; Power Ridges for torque control; mandibular distalization and incisor intrusion; class III elastics (3/16-inch, 3.5 oz, approximately 99 g); aligner force value not numerically reported	SPC every three months	≈ 22 months	Correction of anterior crossbite, reduced mobility and gingival recession, improved alveolar bone morphology, and stable outcomes at three-year follow-up

**Table 2:** Changes in clinical and radiographic periodontal values reported in studies evaluating clear aligner therapy in periodontally compromised patients CAT: clear aligner therapy, SPC: supportive periodontal care, PPD: probing pocket depth, CAL: clinical attachment level/loss, BOP: bleeding on probing, FMPS: full-mouth plaque score, FMBS: full-mouth bleeding score, FMBOP: full-mouth bleeding on probing, PI: plaque index, GI: gingival index, NR: not reported

Author	Study design/follow-up	Periodontal diagnosis	Stability criteria before CAT + SPC	Change in clinical/radiographic periodontal values
Raphaelli Nahás et al. [4]	Case series/12-month periodontal maintenance	Stage III, grade B periodontitis	Periodontal maintenance and oral hygiene instruction every three months post-treatment	Mean values of case 1 and case 2 at baseline and after 12 months, mean PD (mm): case 1: 2.62 → 2.67, case 2: 2.32 → 2.30; mean CAL (mm): case 1: 2.98 → 2.76, case 2: 2.67 → 2.51; plaque (% sites): case 1: 9.25 → 1.23, case 2: 19.23 → 7.05; BOP (%): case 1: 8.64 → 6.79, case 2: 9.61 → 5.12; suppuration: 0 at baseline and final
Ronsivalle et al. [5]	Case report/six months (periodontal phase)	Stage III, grade B periodontitis	SRP before CAT	BOP: 25% → 9%, PI: 40% → 17%, sites with PPD >4 mm: 20 → 3
Zhang et al. [10]	Case report/NR	Chronic periodontitis and pathologic tooth migration; inactive periodontitis	FMPS: 18%, FMBS: 15%, no PPD >4 mm; orthodontic forces applied after three months of systematic periodontal treatment	Clinical: Peer Assessment Rating score reduced by 83% (from 24 to 4), periodontal support maintained and improved; radiographic: buccal bone thickness increased for maxillary incisors; alveolar bone resorption was observed initially in multiple sites but was extensively resolved in the maxillary left lateral incisor
Turatti et al. [17]	Case report/NR	Periodontitis with infrabony defect	SRP before and every eight weeks during treatment	Clinical: Not explicitly detailed in the excerpt, beyond periodontal disease management; post-treatment radiographs clearly showed upper incisor intrusion and a reduction of the infrabony defect
Lee et al. [18]	Case series/NR	Chronic periodontitis with pathologic tooth migration	Periodontal therapy before CAT	Tooth-level reductions in PPD and CAL with improvement in gingival recession and mobility, no pooled mean change reported; radiographic: decrease in infrabony defects (0.8 mm vertically and 0.4 mm horizontally in one case, and 2.1 mm vertically and 0.7 mm horizontally in another case)
Stańdo-Retecka et al. [19]	Case report/three years	Generalized stage IV, grade C periodontitis	Before CAT: PPD: ≤4 mm, FMBOP: <10%, FMPI: 7%, SPC: every two months	Baseline BOP: 54%, FMPI: 46%, post-hygiene FMPI: 24%, one-year PPD: ≤4 mm at all sites, FMBOP: <10%, FMPI: 7%
Tietmann et al. [21]	Retrospective case series (10 patients; 103 defects)/≥1 year	Stage IV (type 2) periodontitis	Successfully completed steps 1 and 2 of periodontal therapy with adequate oral hygiene (FMPS: ≤20%, FMBS: ≤20%); aligner therapy delayed until tooth mobility was <1; SPC started with a strict interval of four weeks up to three months during the whole duration of orthodontic treatment	Mean PPD: 5.40 ± 1.80 → 3.78 ± 1.73 (one year), 5.40 ± 1.80 → 3.73 ± 1.70 (final); pocket closure (≤4 mm): 76%; radiographic bone level gain: +2.13 ± 1.64 mm (one year), +3.02 ± 2.00 mm (final)
Ravera et al. [22]	Prospective pilot study/NR	Stage IV, grade B periodontitis	No residual PPD >4 mm; FMPS: <15%, FMBS: <15%	Statistically significant decrease in PPD (0.66 to 1.76 mm reduction at 12 sites after treatment); improvement in clinical attachment loss, with statistically significant reduction at 10 anterior and 13 posterior sites; slight increase in gingival recession in anterior areas, with worsening at five sites (0.75 to 1.55 mm) and improvement in the posterior area; radiographic: average increase in anterior clinical crown length of 0.2 mm ± 0.18 mm (not statistically significant); significant decrease in posterior area of 0.29 mm (p = 0.049)
Almagrami et al. [23]	Case report/three-year follow-up	Stage III, grade B periodontitis	CAT initiated after periodontal stabilization; patient enrolled in three-monthly supportive periodontal care during CAT	Improved alignment and occlusion with maintained periodontal health; stable clinical and radiographic outcomes over three years

Stage IV Periodontitis and Pathologic Tooth Migration: CAT After Regenerative and Corrective Therapy

The most challenging cases involved stage IV periodontitis with pathologic tooth migration and occlusal collapse, often requiring regenerative periodontal surgery and complex occlusal rehabilitation [3,20]. Across stage IV reports, a consistent protocol emerged. Patients underwent non-surgical therapy, followed by regenerative procedures for intrabony defects and other corrective measures. Orthodontic loading with CAT began only after specific periodontal endpoints: full-mouth plaque/bleeding scores generally below 15%, no moving tooth with probing depths >4 mm, absence of suppuration, and tooth mobility reduced to grade I or less [1,13,21,22]. Typically, aligners started 1-3 months post-regenerative surgery, after initial healing and tissue stability [22,24]. Within this controlled period, stage IV CAT series showed periodontal trajectories similar to those reported for fixed-appliance therapy in comparable patients, but with clear aligners as the active appliance [25].

In a retrospective clear aligner cohort of 10 stage IV periodontitis patients with 103 intrabony defects, the mean radiographic bone-level gain was 2.13 ± 1.64 mm at 12 months and 3.02 ± 2.00 mm at final splinting. Deepest defect analysis showed gains of 3.45 ± 2.33 mm at T1 and 5.01 ± 2.68 mm at T2. The mean probing pocket depth decreased from 5.40 ± 1.80 mm at baseline to 3.78 ± 1.73 mm at T1, remaining stable at 3.73 ± 1.70 mm at T2, with pocket closure in approximately three-quarters of defects [21]. In a prospective pilot study of stage IV, grade B patients treated with CAT post-periodontal therapy, site-level analysis showed statistically significant reductions in PPD and CAL at certain sites, while overall full-mouth indices remained stable, and recession slightly increased [22]. Long-term follow-up in stage IV patients treated predominantly with fixed appliances post-regeneration showed greater gains in larger cohorts. A practice-based cohort of 48 patients with 526 intrabony defects had a mean radiographic bone-level gain of 4.67 ± 2.50 mm after one year and 4.85 ± 2.55 mm after 2-4 years. The mean PPD decreased from 6.00 ± 2.09 mm at baseline to 3.45 ± 1.20 mm at one year and 3.12 ± 1.36 mm at 2-4 years. Pocket closure reached 87%, with only 0.57% tooth loss [26]. Although clear aligners were not used in this large fixed-appliance cohort, the similar trend in periodontal changes to smaller aligner-based series suggests that successful regenerative surgery and rigorous SPC make the choice between CAT and fixed appliances less critical for maintaining/improving bone levels and probing depths in stage IV disease. Case-based CAT reports in stage III-IV periodontitis with PTM further support this. Aligners uprighted/intruded flared incisors, redistributed spaces, reduced overjet, and de-traumatized occlusion post-regeneration/non-surgical therapy. At one-year and three-year follow-up, periodontal indices were stable, with no severe complications attributed to the orthodontic phase. Suboptimal plaque control, not appliance choice, appeared to be the main risk factor for deterioration.

Common Interdisciplinary and Biomechanical Themes

Three consistent themes emerged across stage II-III and stage IV literature. First, clear aligners are always integrated into a stepwise ortho-perio pathway. Risk factor control, non-surgical debridement, corrective periodontal therapy, and SPC are prerequisites. Aligners are introduced as part of step 4, with SPC visits coordinated with orthodontic appointments [1,2,6]. Second, CAT biomechanics are adapted for the reduced periodontium [23]. Digital setups limit movement magnitude per stage, favoring controlled tipping, intrusion, and torque, respecting the residual alveolar bone [10,17,27]. Strategies included reducing attachments on mobile teeth, trimming aligners away from compromised sites, or segmenting treatment. Aligner change intervals were often extended to 14-21 days in fragile cases for slower adaptation [13]. Third, treatment objectives are interdisciplinary. In stage II-III cases, CAT improves cleansability and creates favorable axial positions for restorative solutions. In stage IV post-regenerative cases, aligners redistribute occlusal contacts, re-establish anterior guidance, and stabilize vertical dimension after bone/attachment gain. Success is judged by alignment, stability of probing depths, absence of bleeding/suppuration, and patient satisfaction. Overall, adhering to these interdisciplinary and biomechanical principles allows clear aligners to achieve desired orthodontic and functional changes in stage II-IV periodontitis without compromising periodontal stability.

Synthesis and clinical implications

This narrative review aimed to clarify CAT's role in interdisciplinary management of stage II-IV periodontitis, focusing on PTM and post-regeneration cases. Despite limited and heterogeneous reports, consistent themes guide clinical decision-making and realistically position CAT.

Comparison With Broader Clear Aligner and Periodontal Literature

The review by dos Santos et al. [13] on CAT's impact on periodontal health, focusing on general orthodontic populations, concluded that it is viable in controlled periodontitis, but not superior to fixed appliances for periodontal outcomes [28]. This aligns with the current synthesis. In both general and high-risk populations, plaque and gingival index differences between CAT and fixed appliances are small and clinically less relevant with proper hygiene and monitoring [25,29]. This review contributes by focusing on stage II-IV periodontitis and PTM, emphasizing CAT's integration within a stepwise ortho-perio protocol. The question is not whether aligners "protect" the periodontium more, but whether they can be safely integrated into complex rehabilitation post-periodontal health restoration. Evidence suggests yes, provided strict periodontal preconditions and rigorous maintenance are met, but CAT does not alter fundamental biological rules governing tooth movement in a reduced periodontium.

What CAT Adds in Stage II-IV Disease

In stage II-III or chronic periodontitis with a stable reduced periodontium, CAT's primary contribution appears functional and esthetic. It corrects occlusal sequelae to improve self-care and enable predictable restorative planning. Initiated post-non-surgical therapy in patients with low plaque/bleeding indices and no deep residual pockets at moving teeth, CAT has consistently been associated with stable probing depths, sometimes with small gains in CAL or defect morphology [4,23]. In stage IV disease, especially after regenerative periodontal surgery, CAT's value lies in redistributing occlusal contacts, re-establishing anterior guidance, and stabilizing vertical dimension without extensive fixed appliances in a fragile dentition. Multi-defect cohorts indicate that CAT, introduced 1-3 months after successful regenerative surgery, can support meaningful bone gains and high pocket closure rates, mirroring fixed-appliance outcomes. For motivated, esthetically conscious patients concerned about plaque control, CAT may be an attractive option without compromising periodontal stability. However, no included reports show CAT leading to better periodontal outcomes than carefully planned fixed-appliance therapy in stage II-IV periodontitis [21]. With successful regenerative surgery and intensive SPC, both appliance systems achieve stable outcomes. Long-term success hinges on the quality of periodontal therapy/maintenance, realistic orthodontic objectives respecting the reduced alveolar bone, and sustained interdisciplinary collaboration.

Central Role of the Interdisciplinary, Stepwise Protocol

The most striking theme is the ortho-perio protocol's primacy over appliance choice. In all successful cases, CAT was used only after risk factors were addressed, infection controlled, corrective interventions completed, and patients enrolled in 3-4-monthly SPC. Orthodontic decisions were coordinated with periodontal re-evaluations [1-3,5,6]. Within this framework, clear aligners are part of step 4 rehabilitation. Clinical outcomes in stage II-IV patients suggest that the key determinants for long-term success are as follows: strict plaque/bleeding control, realistic orthodontic objectives respecting the reduced alveolar bone, and sustained collaboration among periodontist, orthodontist, and restorative dentist. Appliance type is secondary to these foundational elements.

Biomechanical and Practical Considerations Specific to CAT

CAT-specific biomechanical themes with practical implications include the following. First, force systems must adapt to the reduced periodontium. Apically displaced centers of resistance in advanced bone loss increase susceptibility to tipping/torque. Digital aligner planning should prioritize small movement increments and controlled tipping/intrusion/torque, and avoid aggressive proclination or extrusion of compromised teeth [17,27]. Strategies to reduce forces on fragile teeth include extending aligner change intervals, reducing attachments on mobile teeth, and trimming aligners away from severely compromised sites [10,30]. In a finite-element study evaluating the optimal orthodontic displacement of clear aligners in mild, moderate, and severe periodontal conditions, stress was shown to concentrate around the cervical region of anterior teeth, which increased with greater tooth displacement and reduced periodontal support, particularly in excessively inclined teeth. To avoid excessive strain at the alveolar crest, aligner displacement was recommended to be below 0.18 mm in stage I (mild), below 0.15 mm in stage II (moderate), and below 0.10 mm in stage III (severe) periodontitis [12]. Second, treatment objectives must be periodontally and orthodontically defined. In many stage II-IV cases, the goal is not ideal occlusion but eliminating trauma, redistributing forces, and creating cleansable contacts. CAT can reposition roots within regenerated bone, close plaque-trapping diastemata, and align teeth for restorative work. Success is measured by stable probing depths, absence of bleeding/suppuration, and patient-centered outcomes, not just marginal alignment gains. Third, retention and long-term maintenance require special attention. A reduced periodontium is vulnerable to pathologic tooth migration relapse. Data on long-term fixed/removable retainer outcomes after CAT in stage II-IV disease are limited, but retainers must be integrated into SPC. The ortho-perio team should jointly decide on retention, with clear instructions on cleaning and periodic reassessment.

Limitations of the Available Evidence

Current evidence has limitations: most reports are single cases or small series from specialist centers, limiting generalizability. Only one retrospective analysis reported on multiple patients post-regenerative surgery with CAT in stage IV disease, lacking a fixed-appliance control. Long-term outcomes beyond 2-3 years are rare. Heterogeneity exists in periodontal staging, aligner systems, case complexity, and reported outcomes. Many studies lack full-mouth charting, standardized indices, or detailed radiographic measurements, hindering comparisons. Compliance with aligner wear and home care is rarely quantified, despite its likely influence on outcomes. Publication bias is a concern; successful cases are more likely to be published. The reassuring picture from existing CAT literature in stage II-IV periodontitis should be cautiously interpreted, not as proof of rare/negligible complications.

Implications for Future Research

Future research should move beyond isolated reports toward well-designed prospective cohorts and, where feasible, controlled comparisons of CAT and fixed appliances in defined stage II-IV periodontitis populations. Standardized reporting of periodontal staging/grading, full-mouth probing depths/attachment levels, radiographic bone measurements, and occlusal parameters would improve interpretability. Incorporating patient-reported outcomes and objective compliance measures would clarify CAT's advantages in adherence or quality of life. Randomized trials may be difficult, but matched cohort designs within specialist centers, using shared stepwise perio-ortho protocols differing only in appliance, could provide clinically relevant information on whether CAT offers incremental benefits in patient comfort, hygiene, or long-term stability. Longer follow-up post-orthodontic treatment, including documentation of relapse, retainer problems, and incident tooth loss, is crucial to understand CAT's true long-term impact in periodontally compromised patients.

## Conclusions

Despite predominantly low-level and heterogeneous evidence, this narrative review supports that clear aligner therapy can be safely integrated into interdisciplinary management of adults with stage II-IV periodontitis when strict periodontal and biomechanical principles are respected. In both stage II-III/chronic periodontitis and stage IV disease post-regenerative/corrective therapy, CAT has been associated with meaningful improvements in occlusion and PTM while maintaining, and sometimes modestly enhancing, probing depths, CAL, and radiographic bone levels. However, available data do not demonstrate CAT's periodontal superiority over carefully planned fixed-appliance treatment in this high-risk group.

When behavioral risk factors are addressed, inflammation eliminated, and patients enrolled in a well-structured SPC, both appliance systems yield stable outcomes. Long-term success depends on quality periodontal therapy/maintenance, realistic orthodontic objectives respecting reduced alveolar bone, and sustained interdisciplinary collaboration. Clinicians should view CAT as one tool within a stepwise ortho-perio protocol for stage II-IV periodontitis, not a standalone or inherently "gentler" option. Judiciously used, after full periodontal stabilization, with adapted biomechanics, and close maintenance, clear aligners contribute to functional rehabilitation, improved cleansability, and esthetic satisfaction in periodontitis patients. These conclusions reflect recurring clinical patterns rather than statistically generalizable outcomes and should be interpreted within the limitations of predominantly case-based evidence. High-quality prospective research is needed to quantify these benefits and clarify if CAT offers clinically meaningful advantages over fixed appliances in long-term management of periodontally compromised patients.

## Appendices

Appendix 1

Table 3 presents the complete search strategies used in the present study.

**Table 3:** Search strategy

Database	Search strategy
PubMed/MEDLINE	((clear aligner[Title/Abstract]) OR (aligner therapy[Title/Abstract]) OR (invisalign[Title/Abstract])) AND ((periodontal disease[Title/Abstract])) OR (periodontitis[Title/Abstract]) OR (periodontally compromised[Title/Abstract]) OR (Periodontal Diseases[Mesh Terms])) AND ((orthodontic[Title/Abstract]) OR (orthodontic treatment[Title/Abstract]))
Scopus	(TITLE-ABS-KEY("clear aligner" OR "aligner therapy" OR invisalign) AND TITLE-ABS-KEY("periodontal disease" OR periodontitis OR "periodontally compromised") AND TITLE-ABS-KEY(orthodontic OR "orthodontic treatment")
Embase	('orthodontic aligner'/exp OR 'invisalign'/exp) AND ('periodontal disease'/exp OR 'periodontitis'/exp) AND ('orthodontics'/exp OR 'orthodontic procedure'/exp)
Google Scholar	"clear aligner therapy" "periodontally compromised" orthodontic
